# A Literature Review on the Development and Creation of Digital Twins, Cyber-Physical Systems, and Product-Service Systems

**DOI:** 10.3390/s23249786

**Published:** 2023-12-12

**Authors:** Michel Fett, Fabian Wilking, Stefan Goetz, Eckhard Kirchner, Sandro Wartzack

**Affiliations:** 1 Institute for Product Development and Machine Elements, Technical University Darmstadt, Otto-Berndt-Straße 2, 64287 Darmstadt, Germany; michel.fett@tu-darmstadt.de; 2 Engineering Design, Friedrich-Alexander-Universität Erlangen-Nürnberg, Martensstraße 9, 91058 Erlangen, Germany; wilking@mfk.fau.de (F.W.); goetz@mfk.fau.de (S.G.); wartzack@mfk.fau.de (S.W.)

**Keywords:** Digital Twin, Cyber-Physical System, development, review, methods

## Abstract

Digital Twins offer vast potential, yet many companies, particularly small and medium-sized enterprises, hesitate to implement them. This hesitation stems partly from the challenges posed by the interdisciplinary nature of creating Digital Twins. To address these challenges, this paper explores systematic approaches for the development and creation of Digital Twins, drawing on relevant methods and approaches presented in the literature. Conducting a systematic literature review, we delve into the development of Digital Twins while also considering analogous concepts, such as Cyber-Physical Systems and Product-Service Systems. The compiled literature is categorised into three main sections: holistic approaches, architecture, and models. Each category encompasses various subcategories, all of which are detailed in this paper. Through this comprehensive review, we discuss the findings and identify research gaps, shedding light on the current state of knowledge in the field of Digital Twin development. This paper aims to provide valuable insights for practitioners and researchers alike, guiding them in navigating the complexities associated with the implementation of Digital Twins.

## 1. Introduction

Digital Twins have received a steadily growing degree of attention in recent years, both from industry and academia. One possible reason for this is a multitude of promising potentials and practical fields of application, such as real-time condition monitoring and the associated predictive maintenance, performance and usage analysis, or collection of information for product development. Extensive knowledge of the system is essential in order to calculate the remaining useful lifetime [1]. In addition to monetisation with existing business models, this concept can also be used to open up new digital business fields, such as pay per stress.

The creation of Digital Twins is a complex interdisciplinary project that requires know-how and experience as well as human and financial resources. For this reason, despite the promising potential, many companies, especially small and medium-sized enterprises (SME), have reservations about introducing Digital Twins. To mitigate this problem, systematic procedures in the form of guidelines and methods can be used to create Digital Twins. There are already a number of publications in the literature that systematically support the creation of Digital Twins. In addition, findings from the related fields of Cyber-Physical Systems and Product-Service Systems can be used to support the creation of Digital Twins.

At the moment, this literature is only available in an unsystematic form and with uneven granularity. This makes an identification or even connection of suitable publications for the specific application difficult. For this reason, this contribution conducts a systematic literature review on the topic of developing and creating Digital Twins (DT), Cyber-Physical Systems (CPS), and Product-Service Systems (PSS). The results are then structured and compared. Although the three different systems—Digital Twins, Cyber-Physical Systems, and Product-Service Systems—are examined, the focus of this paper lies in the technical implementation of Digital Twins.

The goal of this paper is answering the following research questions:

- RQ1: How is the creation of Digital Twins, Cyber-Physical Systems, and Product-Service Systems considered in the literature and what research directions exist?
- RQ2: In what areas is further research needed to support direct utilisation for the creation of Digital Twins, Cyber-Physical Systems, and Product-Service Systems?

## 2. Materials and Methods

This section first introduces the fundamentals of Digital Twins. The concepts of Cyber-Physical Systems and Product-Service Systems are described as well. This is followed by a brief presentation of existing literature on the topic of creating Digital Twins, Cyber-Physical Systems, and Product-Service Systems. Finally, the study design for the research of this contribution is described.

### 2.1. Fundamentals and Definitions of Digital Twins

If a mechanical system is extended by electric or electronic components, and thus the range of functions is extended, it is called a mechatronic system. These components can be sensors, actuators, and microcontrollers and enable the system to be controlled on the basis of loops [2,3,4].

Mechatronic systems are the foundation for Cyber-Physical Systems (CPS). In this context, mechatronic systems are further developed by integrating embedded communication units that enable the system to connect or communicate with other systems and the Internet of Things and interact with its environment. For example, the behaviour of the system can be changed in response to the communication and interaction [2,4].

The communication capability of CPS is a necessary premise of the Digital Twin concept. The Digital Twin concept follows a more model-based approach. A Digital Twin is a digital representation of a product instance [4,5]. The product instance can be a physical product or a service and is then referred to as a physical twin. The Digital Twin is able to model the behaviour of the physical twin and thus enables conclusions and predictions through calculations and simulations. For this purpose, there is a bidirectional connection between the physical and Digital Twin for the exchange of information. Operational data from the physical twin is transferred to the Digital Twin, where it feeds the models. Operating data can be recorded using classic sensors which are integrated into the system or the environment. Alternatively, sensor-integrated machine elements [6,7], sensor-integrated design elements [8], or soft sensors [9] are also suitable for acquiring operating data. Sensors can either be taken into account during the development of the physical twins or retrofitted [10]. The results of the calculations and simulations are fed back into the physical space or directly to the Digital Twin. The fields of application for Digital Twins are very diverse. Some examples are predictive maintenance in manufacturing and stress identification in the context of agriculture, smart cities, supply chain optimisation, or healthcare diagnoses in humans [11].

**Figure 1:**
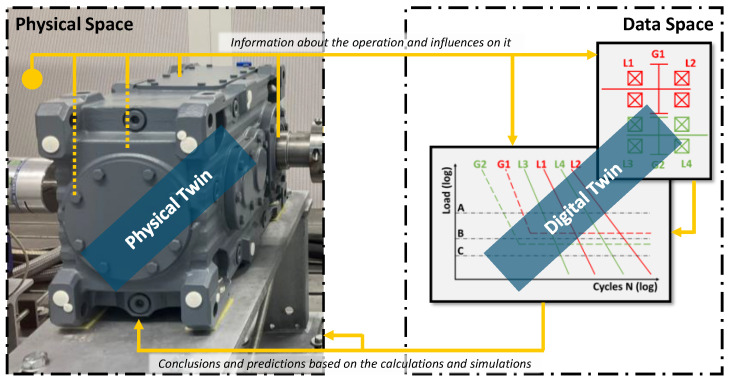
Schematic representation of the Digital Twin concept.

Figure 1 demonstrates the Digital Twin concept using the example of a two-stage industrial gearbox. The physical twin is located in the physical space. Data are collected with sensors in or on the gearbox or in its environment. The data are then transferred from the physical space to the data space, where they feed behaviour-describing models of the gearbox. These can be, for example, RUL calculations of individual components. Here, the Wöhler curves of the components, such as bearings (L) and gears (G), can be used. The results are then transferred from the data space back to the physical space. There, they can be used to display results or recommendations for action. If the data flow does not end on the physical space but instead flows directly into the physical twin, its operation can be adapted.

The ability to observe the physical twin and make conclusions and predictions about its behaviour opens up a range of new possibilities, such as predictive maintenance, performance analysis, adjustment of operating modes, or prevention of misuse. In this way, not only the product itself can be offered to a potential customer, but also further services. In this context, the term Product-Service System (PSS) is used. In addition to the product and the services, a PSS also requires a network of stakeholders and supporting infrastructure [12,13,14].

### 2.2. Existing Literature Reviews

There are several review and survey articles that analyse and categorise procedures for the development of DT, CPS, and PSS. These were identified and analysed as part of an initial literature search and are listed in Table 1. Most reviews are dedicated to the development of CPS. While Horvath et al. [15] provided a more general overview of the state-of-the-art, Korotunuv et al. [16], Liu et al. [17], and Quadri et al. [18] focused on modelling and design methodologies for CPS. The two reviews by Mohamed et al. [19,20] are particularly noteworthy due to the scope of the literature examined. They provided a broad overview of the CPS literature. Wu et al. conducted two literature reviews. One focused on design and implementation methods [21] and the other on concept and engineering development [22].

In comparison, there are only a few existing review papers for the creation of Digital Twins. Pater et al. [23] contrasted the topic of Digital Twins with the topics discussed in the literature. Adamenko et al. [24] compared modelling methods for Digital Twins.

Reviews also exist in the literature within the area of Product-Service Systems. Qu et al. [25] outlined the state-of-the-art with regard to design, evaluation, and operation methodologies for PSS, while the other reviews focus, in particular, on methodologies for creation. Mendes et al. [26] analysed PSS design processes, while Müller and Blessing [27] compared different approaches for product and service development. Gräßle et al. [28] analysed procedures in terms of characteristics related to the design process and results, PSS development goals, and PSS-specific and non-specific characteristics. Both Clayton et al. [29] and Haber and Fargnoli [30] contrasted the methods and methodologies from the literature with criteria from the creation phases of the PSS. Annamalai Vasantha et al. [31] created a maturity model to visualise the current state of development of PSS.

**Table 1:** Overview of existing literature reviews.

Source	Author	Title	System	Literature
[24]	Adamenko et al.	Review and Comparison of the Methods of Designing the Digital Twin	DT	3
[29]	Clayton et al.	Evaluating Existing Approaches to Product-Service System Design	PSS	12
[28]	Gräßle et al.	Vorgehensmodelle des Product-Service Systems Engineering	PSS	11
[30]	Haber and Fargnoli	Designing Product-Service Systems: A Review Towards a Unified Approach	PSS	20
[15]	Horvath et al.	Compositional Engineering Frameworks for Development of Smart Cyber-Physical Systems: A Critical Survey of the Current State of Progression	CPS	19
[16]	Korotunov et al.	Cyber-Physical Systems Architectures and Modelling Methods Analysis for Smart Grids	CPS	10
[17]	Liu et al.	Characteristic, Architecture, Technology, and Design Methodology of Cyber-Physical Systems	CPS	30
[26]	Mendes et al.	Product-Service System (PSS) Design Process Methodologies: A Systematic Literature Review	PSS	246
[19]	Mohamed et al.	A Systematic Literature Review on Model-Driven Engineering for Cyber-Physical Systems	CPS	187
[20]	Mohamed et al.	Model-Driven Engineering Tools and Languages for Cyber-Physical Systems—A Systematic Literature Review	CPS	187
[27]	Müller and Blessing	Development of Product-Service Systems—Comparison of Product and Service Development Process Models	PSS	7
[23]	Pater and Stadnicka	Towards Digital Twins Development and Implementation to Support Sustainability—Systematic Literature Review	DT	20
[25]	Qu et al.	State-of-the-Art of Design, Evaluation, and Operation Methodologies in Product-Service Systems	PSS	258
[18]	Quadri et al.	Modelling Methodologies for Cyber-Physical Systems: Research Field Study on Inherent and Future Challenges	CPS	58
[21]	Wu et al.	Cyber-Physical Production Systems: A Review of Design and Implementation Approaches	CPPS	25
[22]	Wu et al.	Concept and Engineering Development of Cyber-Physical Production Systems: A Systematic Literature Review	CPPS	100
[31]	Annamalai Vasantha et al.	A Review of Product-Service Systems Design Methodologies	PSS	22

## 3. Study Design

In this work, the similarities and connections between DT, CPS, and PSS were considered in order to create an overarching review of the corresponding procedures described in the literature. The review was conducted in accordance with the PRISMA approach for systematic literature research [32,33]. For this purpose, a search string was created that included the terms DT, CPS, and PSS, as well as corresponding synonyms. Building on the findings of the reviews examined in Section 2.2, the search string was further supplemented with synonyms for “procedure” and “development”. The word search categories were linked by use of the Boolean operators AND and OR. Since the literature found was screened for suitable titles as the first step, the search terms were deliberately limited to the title. Thus, the resulting search string was as follows:

TITLE ("digital twin*" OR "virtual twin*" OR Avatar* OR "Digitale* Zwilling*" OR "Virtuelle* Zwilling*" OR "Cyber Physical System*" OR "Cyber-Physical-System*" OR CPS OR "Cyber Physical Production System*" OR "Cyber-Physical-Production-System*" OR CPPS OR "Cyber Physical Twin" OR "Cyber Physischer Zwilling" OR "Product Service System*" OR "Produkt Service System*" OR "Product-Service-System*" OR "Produkt-Service-System*") AND TITLE(*method* OR *approach* OR *framework* OR *strateg* OR "process model" OR *schema* OR *scheme* OR systema*) AND TITLE(creat* OR develop* OR implement* OR model* OR deploy* OR design*)

This search string was used to identify suitable literature in five databases for scientific literature. These databases were Inspec, ProQuest, Scopus, TEMA, and Web of Science. After duplicates were eliminated from the totality of articles, the remaining articles were screened, first by title and then by abstract. The remaining articles were then screened for their suitability for the scope of this paper. The criteria applied were as follows:

- The article should describe the creation of the DT and not only its usage.
- The technical and technological aspects of the creation were in the focus. Articles that focused purely on organisational or economic aspects were discarded.
- The DT should also be in a technical context. DT of, e.g., humans, buildings, or agricultures, were neglected.

**Figure 2:**
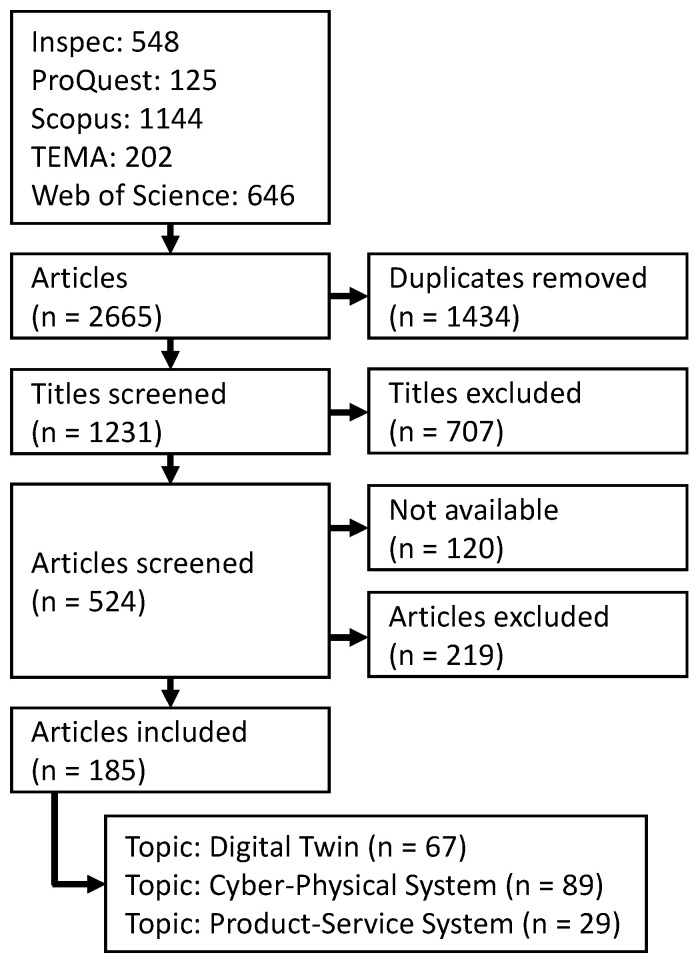
Schematic representation of the research approach.

The procedure, in accordance with the PRISMA approach, is schematically shown in Figure 2.

**Figure 3:**
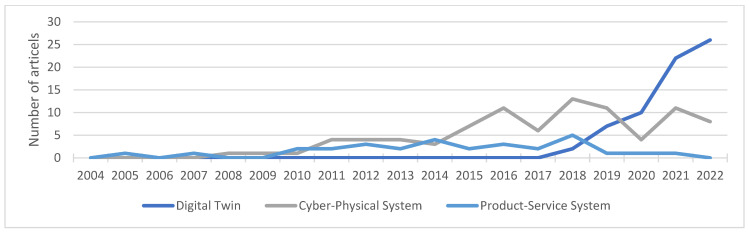
Number of literature articles found, sorted by systems and the years of publication.

A breakdown of the literature found by date of publication revealed clear trends. The first publication on the topic of DT examined in this contribution was published in 2018. Since then, however, the number of publications on this topic has risen sharply and continues to show an upward trend. Publications on the topic of CPS showed a less pronounced upward trend between 2008 and around 2016. Since then, the number of publications in this area has stagnated at a moderately high level. In the field of PSS, a slight increase can be observed between 2010 and 2018 and, since then, a slow decrease in the number of publications. The trends are shown in Figure 3.

**Figure 4:**
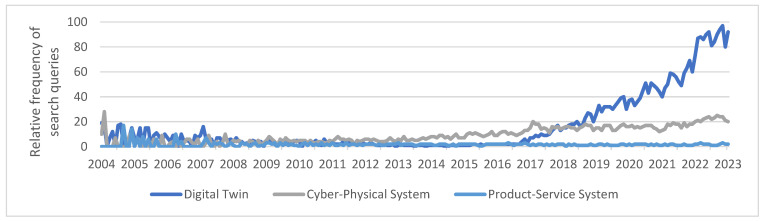
Development of search queries on Google.

When these trends were compared with the number of search queries on the Google search engine, large similarities became apparent. In the context of this article, this was performed using the “Google Trends” application [34] with the topics “Digital Twin”, “Cyber-Physical System”, and “Product-Service System”. Figure 4 shows the results of the development of search queries worldwide over the last 20 years. The value 100 indicates the maximum number of search queries, and the absolute values were not determined.

While occasional peaks can be observed for all three search terms in the period from 2004 to 2008, the number of search queries for the topic “Digital Twin” has rapidly increased since 2017. The interest in this topic continues to increase every year. The topic “Cyber-Physical System” has also been searched more frequently since around 2010, although the growth observed here is nowhere near as rapid as that for Digital Twins. Finally, no growth beyond statistical noise can be observed for the search term “Product-Service System”. Overall, the number of search queries here is very low compared to Digital Twins and Cyber-Physical Systems.

## 4. Results

The articles cover aspects that can be divided into three categories: “holistic approaches”, “architecture”, and “models”. As described in the Introduction Section, the focus of this article is on the creation of Digital Twins. Even though the literature is spread over the three different systems—Digital Twins, Cyber-Physical Systems, and Product-Service Systems—in the following, the term Digital Twins will be used primarily.

The category “holistic approaches” includes procedures that describe the creation of Digital Twins across different domains. These are not limited to one domain, and often do not distinguish between them. Instead, they include aspects of modelling, the creation of an IT infrastructure, and/or the integration of appropriate sensors, simultaneously. Some publications also consider other domains, such as economic aspects.

The second category, “architecture”, deals purely with aspects that are necessary for data transmission. Here, software and hardware aspects are considered, which enable data to be transferred from the physical space into the data space and processed there.

**Figure 5:**
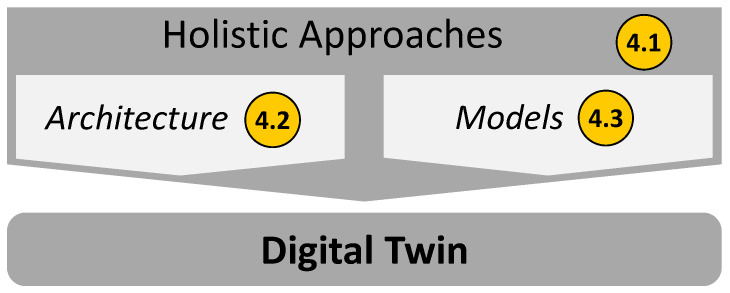
Context of the categories of this contribution.

The third category, “models”, includes literature that deals with the creation of models. It considers the necessary steps in the modelling process, but also the modelling scope and modelling types. Figure 5 shows how these categories interact in the context of the Digital Twin and refers to the corresponding sections in this contribution.

**Figure 6:**
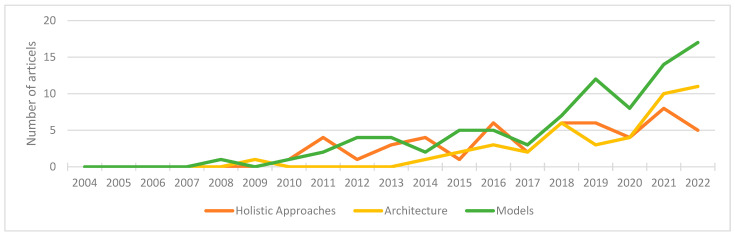
Number of literature articles found, sorted by domains and the years of publication.

If the number of publications classified into these three categories is considered over the year of publication, trends can be identified. For all categories, a steady increase in publications can be observed over the years. Publications on holistic approaches and models started as early as 2010, while publications on architecture were only published from 2014 onwards, with only a single exception in 2009. Furthermore, the number of publications on models started to increase more rapidly in 2017. This also corresponds to the year in which the number of publications and Google searches on the topic of Digital Twins began to significantly increase. Figure 6 shows the trends plotted over the years. The literature cannot always be clearly classified into one of these three categories, but it addresses several topics in some cases. For this reason, the sum of publications in Figure 6 differs from the sum of publications in Figure 3.

**Figure 7:**
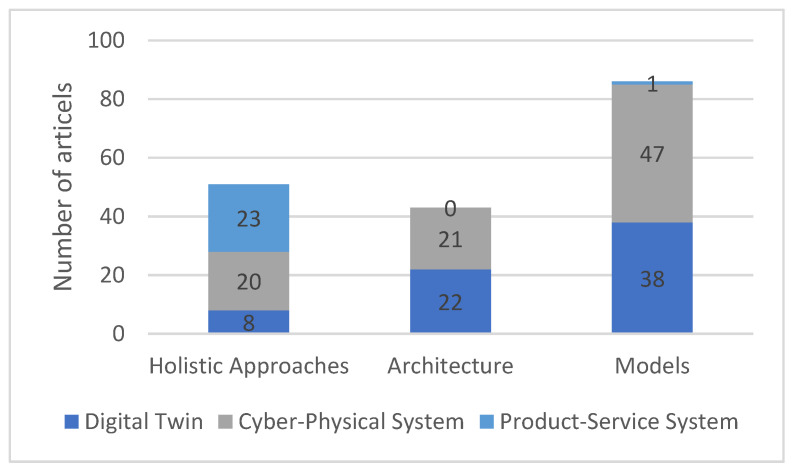
Categorisation of the studied literature into the three main categories.

Figure 7 illustrates the sum of the literature articles in the three categories: “holistic approaches”, “architecture”, and “models”. It is further divided by which system (DT, CPS, or PSS) is treated by the corresponding literature. Publications that deal exclusively with a literature review were not included here. The CPS literature treats the categories “holistic approaches” and “architectures” to a comparable extent. However, models are addressed here more than twice as often as architecture or holistic approaches. A similar relationship is seen in the DT literature between the architecture and the models categories. However, the holistic approaches are treated here more rarely. Literature dealing with the creation of PSS deals with holistic approaches, with only one exception.

### 4.1. Holistic Approaches

There are several publications that deal with the creation of Digital Twins and CPS. On the one hand, the publications in this direction can be divided into the formulation of criteria for appropriate approaches and analysis of existing methodologies. On the other hand, there is literature that presents specific approaches, either as a modification of existing approaches or entirely on its own.

**Figure 8:**
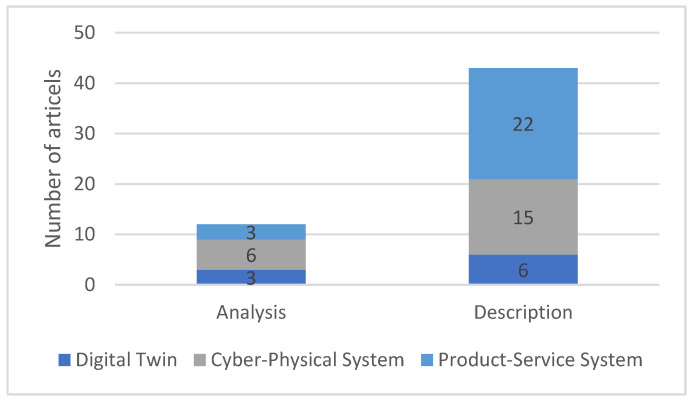
Breakdown of the literature on holistic approaches into subcategories.

Figure 8 shows the breakdown of the literature in the “holistic approaches” category into the two subcategories of “analysis” and “description”. In the subcategory “analysis”, existing and largely established development approaches were examined regarding their suitability for creating Digital Twins. Among other things, criteria were derived that the potentially suitable approaches must fulfil. The subcategory “description” includes literature that introduces and describes modified or completely new holistic approaches. This often builds on existing approaches.

The literature may address both subcategories and can, therefore, be sorted into both. For this reason, the sum of the literature in the subcategories is not equal to the corresponding quantity in Figure 7. For both DT and CPS, about twice as many publications described a (new) procedure compared to those that analysed existing ones. Publications dealing with PSS were almost exclusively devoted to the description of the procedure.

#### 4.1.1. Literature with a Focus on the Analysis of Holistic Approaches

First, the literature on formulating criteria for appropriate approaches and analysing existing methodologies was considered.

Amrani et al. [35] examined two general paradigms for the development of systems: a formalism-oriented paradigm in the form of an object orientation and a workflow-oriented paradigm in the form of agile development. They transferred the findings into a metamodel for describing paradigms.

Perno and Hvam [36] developed a framework to help determine a suitable scope for a Digital Twin. They focused on the use cases and stakeholders of the Digital Twin. 

Both Zheng et al. [37] and Aigner and Khelil [38] formulated various criteria that a procedure must fulfil in order to be suitable for the development of CPS. Zheng et al. [37] used the criteria to evaluate different methods from the areas of V-model-based design methods, MBSE, and agile methods. Aigner and Khelil [38] then compared these criteria with model-based methodologies and cyber-space domain concepts and created concepts for a methodology blueprint for the CPS architecture and an engineering process for CPSs. Chauhan et al. [39] identified the different aspects that need to be considered in the development of CPSs, namely, domain concerns, functional concerns, platform concerns, and deployment concerns. Comparable to this, Jeschke and Grassmann [40] elaborated the necessary requirements and development steps for the development of a Digital Twin in the context of German rail transport. 

Riedelsheimer et al. [41] compared the suitability of different development approaches for the development of Digital Twins. In a similar way, Thammarak [42] investigated the suitability of agile development procedures for CPS. Agile procedures for the development of CPS were also examined by Schuh et al. [43], who furthermore compared three conventional and hybrid procedures with it. Schuh et al. presented a procedure that characterises the specific, individual use case and, based on this, proposes the most suitable development process.

In the context of PSS, existing approaches were also used and evaluated regarding their suitability. While Sadek and Köster [44] evaluated existing approaches of multidisciplinary development, Pezotta et al. [45] considered the waterfall model, V-model, and spiral model. Qu et al. [25] examined various modelling techniques, visualisation methods, modularity methods, and TRIZ.

#### 4.1.2. Literature with a Focus on the Description of New Holistic Approaches

Several publications proposed integrated procedures for the creation of CPS or DT. Both Riedelsheimer et al. [41] and Lowenstein and Mueth [46] adapted the established V-model so that it could be used to create Digital Twins.

However, the majority of the procedures presented in the literature are described step-by-step without explicitly referring to existing approaches. Hehenberger et al. [47] considered three different disciplines of CPS (physical processes, computations, and integration of computations and physical processes) and related them to the early design process (consisting of the conceptual design phase and system modelling phase).

Slomka et al. [48] presented a generic design flow of CPS. This consists of five central steps. First, (1) the requirements are defined. Based on this, (2) the system is specified and designed. This is further specified during (3) the component specification. These are then (4) combined into a system architecture. Finally, (5) a comprehensive constraint analysis takes place. 

Kofanov and Sotnikova [49] also presented a five-step procedure for creating a CPS with a digital counterpart using the example of a spacecraft. First, (1) the physical part of the CPS is developed and (2) the digital counterpart of the physical processes is created. Then, (3) the distribution of the physical variables is determined and (4) the installation position of the sensor is defined based on load cases. Finally, (5) a database is created with which future sensor values can be compared.

Jarvis et al. [50] presented a five-step procedure for the creation of CPS. Starting with specifying the (1) requirements, the (2) physical architecture, and the (3) agent team architecture. Then, (4) the two architectures are mapped and (5) the CPS is finalised.

Merlo et al. [51] presented a procedure consisting of a total of 16 steps, which were divided into 3 phases: exploration, user-centred, and development.

Wu et al. [21,22] categorised the results of a literature review on CPPS into two stages: the concept development stage and the engineering development stage. The former consists of three phases: the (1) needs analysis phase, (2) concept exploration phase, and (3) concept definition phase [22]. The engineering development stage, in turn, is based on the 5C Architecture [21,22].

Rogall et al. [52] and Francalanza et al. [53] also presented procedures for the systematic development of CPPS. Rogall et al. [52] start by (1) defining the system and identifying the relevant variables. Then, (2) the understanding of the system is developed and the relationships between the variables are considered. Next, (3) the specific objectives and stakeholders are considered, and a use case is derived. (4) The necessary data streams and IT elements are built and the CPPS setup is created before (5) the system is evaluated.

Francalanza et al. [53] also considered a modularisation of the CPPS. For this, the (1) requirements are clarified, followed by (2) the selection of technical solutions. Then, (3) module concepts are generated, (4) evaluated, and (5) improved. 

Nogueira de Andrade et al. [54] presented a six-step methodology for the creation of Digital Twins. This consists of (1) obtaining data, (2) model creation, (3) communication establishment, (4) configuring real-time simulation, and (5) development of the control logic and (6) the graphical interface.

Psarommatis and May [55] presented a DT design methodology with seven steps. These steps (1) define the purpose of the DT and (2) identify the asset or process to be represented. Then, (3) the right technologies are chosen and (4) the input and output parameters of the DT are determined. This is used to (5) define the characteristics of each parameter. Finally, there is a (6) performance testing and (7) deployment of the DT.

Jensen et al. [56] presented a ten-step procedure for a model-based design (MBD) for CPS. First, (1) the problem is described and (2) the physical process is modelled. Then, (3) the problem is characterised and (4) a control algorithm is derived before (5) models of computation are selected. Further, (6) the hardware is specified and (7) the computation problem is solved by a simulation approach. Finally, (8) the device is constructed and (9) the software is synthesised. The system (10) is verified, validated, and tested.

Some authors described procedures that cannot easily be presented in lists of individual steps, such as Wu et al. [21,22]. Julien and Martin [57,58], as well as Ballarino et al. [59], followed the 5C model when creating the CPS or DT.

Based on the analysis of different approaches by science and industry described above, Aigner and Khelil [38] developed a methodological blueprint of the CPS architecture consisting of different layers, as well as an engineering process for CPSs.

Rivzi and Chew [60] looked at the state-of-the-art on the creation of CPSs and on CPPSs in general and derived a procedure for the creation of CPPSs. This procedure consists of a detailed description of numerous interconnected individual steps, so that the description of this procedure would go beyond the scope here.

Specific tools for the creation of CPS were presented by Rakov [61] and Larsen et al. [62]. Rakov [61] first identified 15 main attributes of a CPS. For each of these attributes, they presented some implementation emotions, resulting in a morphological box. Larsen et al. [62] presented a prototype of an online platform that includes a sandbox for creating CPS. Within the framework of model-based design (MBD), users can select components from the classes’ models, tools, and operating systems.

The approaches for creating PSSs focus, to a large extent, on organisational and economic aspects. Since the focus of this contribution is primarily on technological aspects, many PSS approaches are, therefore, only relevant to a limited extent. However, a large area of intersection occurs in requirements’ identification involving stakeholders [12,25,63,64,65,66,67,68,69,70,71,72,73,74,75,76,77]. Based on this, some authors created the functions and concept of the PSS [12,66,67,69,72,73,74], which also needs to be implemented from a technological point of view and is, therefore, also relevant to this contribution. In addition, Arioli et al. [63] considered conceptualisation of the infrastructure and network and, as part of a design step, data flow, adaptation of the product, IT architecture, and sensor technology. Nemoto et al. [70] transferred the customer needs into a PSS function model, from which required actors are derived. Apostolov et al. [78] developed services using the V-model and RFLP concept to model use cases. Marques et al. [79] presented approaches for product development and service development and put them into contrast. Sadek and Köster [44] created an integrated approach with micrologic and macro-logic based on their evaluation of existing approaches. Tran and Park [80,81] presented, in two publications, an approach to adapt PSS design methodologies with respect to appropriate criteria.

### 4.2. Architecture

The architecture of a DT describes the necessary structure for required key technological elements. The term architecture is often used synonymously with the terms framework or IT infrastructure. In order to create a uniform understanding, these terms are defined in Table 2. In the context of this article, only the term architecture is used, even if different articles have partly used other terms in a synonymous way.

Different types of architectures for a Digital Twin are available. For example, Diaz et al. [85] drew a comparison between a monolithic architecture (consisting of data, business logic, and web) and a microservice architecture. Dumitrache et al. [86] presented a generic architecture of an enterprise in the context of CPS, also considering business aspects, for example.

The most common classification is the general classification into physical space and cyber space (also called virtual or cloud space/layer) [87,88,89,90,91,92,93,94,95,96,97]. Some authors explicitly mention the communication between these two spaces [89,91,94,95,97]. Several authors present a more fine-grained classification of infrastructure. These finer granular considerations are compatible with the general classification into physical and cyber space and complement it in many places or substitute it with more detailed considerations. The publications that do not explicitly mention the general classification are nevertheless fully compatible with it. In the following, the finer granular considerations are integrated into the general classification and assigned to the literature.

**Figure 9:**
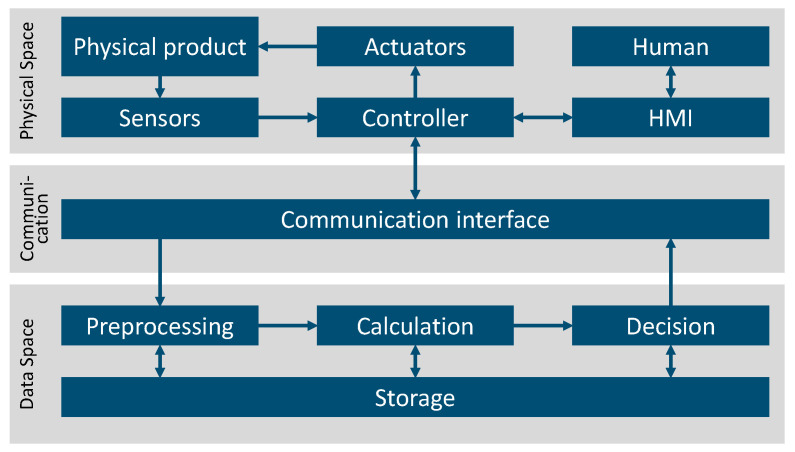
Structure of the overall architecture into physical space, communication, and data space.

Figure 9 shows the division between physical space, data space, and the communication in between. Further, the more detailed subdivision, which is proposed by some authors in the literature, is sorted here.

**Figure 10:**
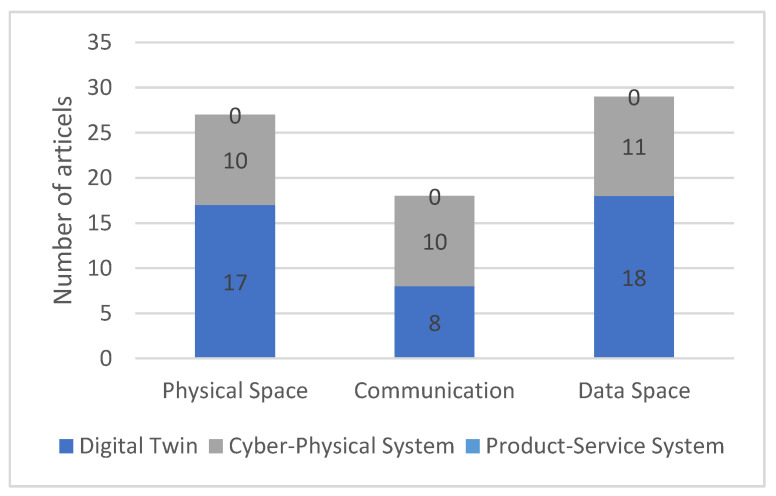
Breakdown of the literature on architectures into subcategories.

Figure 10 shows the breakdown of literature in the “architecture” category into the three subcategories: “physical space”, “communication”, and “data space”. The subcategory “physical space” deals with hardware components that are located on, in, or around the physical twin. The “communication” subcategory includes aspects that serve the transmission of data between the physical space and the data space. Finally, the subcategory “data space” deals with aspects of data processing in the Digital Twin itself.

The literature may address more than one subcategory and can, therefore, be sorted into multiple categories. For this reason, the sum of the literature in the subcategories is not equal to the corresponding quantity in Figure 7. Literature on CPS addresses all three categories equally often. In the context of DT, physical space and data space are addressed about equally as often, but the communication in between is addressed only around half as often.

#### 4.2.1. Physical Space

On the physical side, only a few details are discussed in the publications, and the focus is often on cyber space. For this reason, many authors only mention the physical device and/or the data collection, for example, through sensors. Only a few publications go beyond these core components.

One “component” in physical space is humans, who can act as users or operators [87,88,95]. The users are interconnected with the technical system via a user interface [86,95]. The second, and at the same time, the key component in the physical space is the physical product itself [87,88,90,92,93,95,98,99,100,101,102,103,104,105,106,107,108,109]. The physical product is equipped with components for data acquisition, primarily integrated sensors [86,87,88,89,90,92,93,95,98,99,100,101,102,103,110,111,112]. However, external data sources or operational data can also be used [102]. In general, the data can come from the entire product life cycle [113,114]. Babiceanu and Seker [115] explored the use of a Big Data approach, where the information sources can take different forms. To control the physical product, some authors mentioned the use of actuators [87,89,92,95,98,112]. The sensors and actuators are connected on the physical side to a control unit such as a PLC (Programmable Logic Controller) [87,89,90,103,112,116].

#### 4.2.2. Communication

In order to be able to exchange data between the physical space and the cyber space, a corresponding network is necessary, which establishes a bidirectional connection between the physical and virtual aspects [87,88,90,92,95,97,101,108,109]. The communication takes place via interfaces that connect the physical space and the cyber space [89,91]. Francalanza et al. [53] mentioned a bus module with an IoT gateway and the OPC/UA communication standard. The OPC/UA communication standard was examined in more detail by Liu et al. [117], who compared it with MTConnect. Another standard that is frequently mentioned in the literature is MQTT [116,118]. Mishra and Ray [90] listed a sum of other communication possibilities. Shin et al. [119] presented an approach for creating a low-cost communication system for CPS. They built on an existing commercial communication infrastructure and used a middleware gateway to connect it to the physical system. Both Li et al. [120] and Bernady et al. [121] analysed further necessary sub-aspects and components and developed the necessary requirements.

#### 4.2.3. Data Space

Often, the data cannot be processed directly but must be pre-processed [110,122]. This is also referred to as the use of a conditioner [98]. Pre-processing involves a preliminary analysis of the data [95,110]. On the one hand, the data are converted and/or compressed [99,102] and, on the other hand, useful information and features of the data are extracted [99,102,111].

The selected data or the information and features are then used for the calculations and/or simulation [86,90,91,92,93,98,99,102,103,110,112]. For this purpose, models are used that represent the physical product [88,95,100,104,108,109,111]. These models can be, for example, analytical simulations [89,95,105,122], statistical models [105], mathematical functions [107], or machine learning models [90,105,110,111]. A more detailed consideration of the models follows in the next section of this contribution.

The evaluation and utilisation of the results of the previous calculations and simulations are highly scenario-specific [90,107,122]. Various authors speak in this context of service [103,104,108,109] or application [87,88,90,92,101,102,103,106]. Chen et al. [88] and Jia et al. [101] provided a number of examples for this. In the following, the services are considered, which are also mentioned by the other contributions. It is possible to recognise and react to events based on the simulation and calculation results [99]. For this purpose, it is possible for the system to make decisions within a spectrum [88,95,99,100]. This allows an intelligent operation of the system [88]. Furthermore, a real-time monitoring of the system and the performance is possible [93,95,102,103,106], which can be used for health management [88,101,106]. It is also possible to make predictions about the system [93,102]. The models are to be connected via a suitable architecture, as considered by Lektauers et al. [123] and Wang and Jin [103].

The data are stored in a database and can be retrieved from it [91,95,99,102,111,124,125]. Li et al. [102] and Eyre et al. [116] mentioned an SQL database as a possible implementation. The database is connected to all positions in the data processing chain [108,109]. The stored data can be put in physical space [93], the fog [98,99], or the cloud [87,98,101,103,104].

### 4.3. Models

The models describe the behaviour of the physical product and are a key element of the Digital Twin. In the context of Digital Twins, the models are so present that the two terms are used synonymously by some authors. The literature can be further divided into three subcategories: “procedure steps”, “modelling scope”, and “model type”. The subcategory “procedure steps” describes the individual steps that must be carried out to create the models. In the “modelling scope” subcategory, the modelling scope and, thus, what is to be represented within the framework of the models, is described. The third subcategory “model type” contains different types of models. Furthermore, modelling languages or simulation software, for example, are discussed.

**Figure 11:**
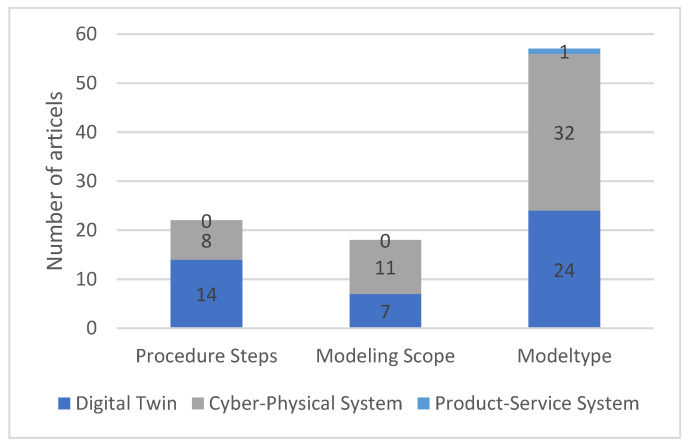
Breakdown of the literature on models into subcategories.

Figure 11 shows the breakdown of the literature in the “models” category into the three subcategories. The literature can address more than one subcategory and can, therefore, be sorted into multiple categories. For this reason, the sum of the literature in the subcategories is not equal to the corresponding quantity in Figure 7. Both the procedure steps as well as the modelling scope are equally covered by the literature on CPS and DT. In comparison, the literature on model type is much more represented.

#### 4.3.1. Procedure Steps

**Figure 12:**
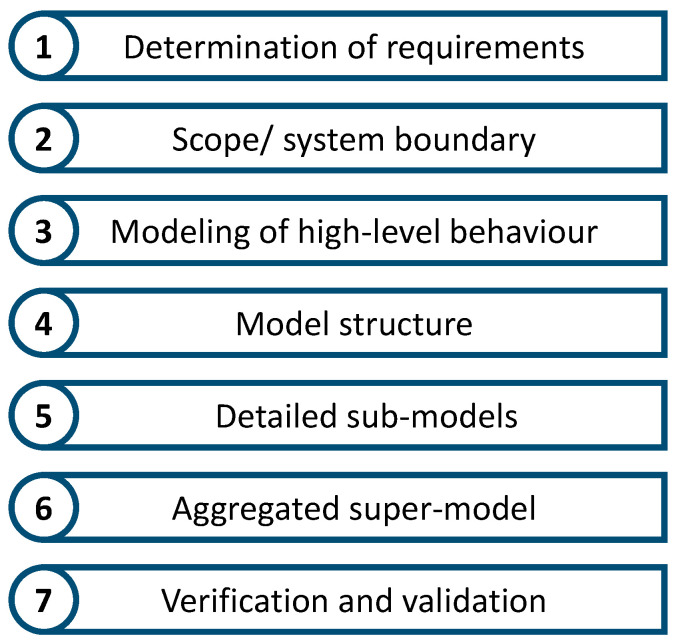
Overview of the procedure steps in the creation of models.

The creation of models can be carried out by applying existing engineering approaches. Lopez and Akundi [126] used model-based system engineering (MBSE) approaches, while Michael and Wortmann [127] followed model-driven software engineering (MDSE) techniques. In contrast to this, various authors presented the necessary steps to create appropriate models. Existing differences can be explained with different granularities and/or scopes. Figure 12 shows the sub-steps described in the literature, which are explained in the following.

The modelling process begins with preparation [128] and **requirements’** identification [129,130,131,132]. For this, the problem to be solved is described and analysed. This results in requirements and necessary specifications for the models [100,133].

The intended use of the Digital Twin determines the **scope** of the Digital Twin and the modelling scope [134,135]. This can be supported by identifying suitable observable objects [136] and is separated from the environment by defining a system boundary [137].

Based on the modelling scope, the **higher-level behaviour** of the physical product can be modelled [129,137,138,139,140]. In the understanding of Gao et al. [141], this corresponds to the representation in the problem domain. Alternatively, or complementing the modelling of the higher-level physical behaviour, a macroscopic **model structure** or architecture can be created for the subsequent detailed models [133,140,142,143,144].

Based on the created macroscopic structure or architecture, or in order to describe the higher-level behaviour of the physical product on a detailed level, more **detailed sub-models** have been created to represent individual aspects of the behaviour. These are specific models for calculation and simulation [129,143] and can be achieved in different ways depending on the model. The subdivision into sub-models can be performed via individual domains [133,141] or via the component and subsystems [137,139]. These represent the individual characteristics, detailed features, and parameters of the system [134,138,145] and can be linked to real operating data [134,146]. This corresponds to the description in a solution domain. Furthermore, the addition of semantic annotations is possible [136,144]. The detailed modelling requires a choice of computing models and a determination of the hardware to be used [129].

The detailed sub-models are then deployed and **aggregated into a super-model** [129,133,134,136,138,139,140,141,144,145]. In the software context, this can be described as software synthesis or code generation [129,141].

In the final step, **verification**, **validation**, and/or testing takes place [128,129,130,140]. Documentation is also necessary [146].

#### 4.3.2. Modelling Scope

Wan et al. [147] combined model-driven engineering (MDE) with component-based design (CBD) for the design of CPPS. They used one model (type) that can be used for different domains. Apart from this, however, several different model types were used in the rest of the considered literature, which are linked with each other. As described in the previous section on cyber space, one possible breakdown is into a descriptive model and a decision-making model [88,95,99,100]. This division was also used by Luo et al. [148,149] and Alessandro Pinto [150]. A large number of alternative model scopes can also be found in the literature. The most mentioned are as follows:

- System model [100,151,152]
- Components, such as the physic and cyber components [152,153,154]
- Process model [100,152,155]
- Behaviour model [156,157,158]
- Simulation model [100,155]
- Rule model [153,157]
- Data transfer model [153,154]
- Environment model [159,160].

In addition to these model types, others are mentioned in some of the reviewed literature. However, due to the fact that these are only mentioned once in the literature, they are not all listed, and instead, reference is only made to the named sources.

#### 4.3.3. Model Type

As already described in the section on cyber space and modelling scope, Digital Twins can include a variety of different models. Adamenko et al. [24] differentiated between two key modelling approaches, which resulted in two different types of models: data- and system-based models. Data-based models are created on the basis of extensive datasets, for example, using machine learning approaches. In contrast, system-based (or physics-based) models use known relationships to describe behaviour. The relationships can be represented in the form of simulation models, mathematical and statistical models, or logical models. Furthermore, defined modelling languages can be used. This requires a high degree of system knowledge. There are already a large number of commercial and industrially used software solutions for modelling simulation models, which may be a possible explanation for why these are not the focus of the literature. Instead, the focus is on modelling via modelling languages, mathematical, and logical modelling, but also model creation via machine learning.

In the literature on modelling languages, Lichen Zhang is particularly noteworthy, with a large number of publications. In several papers [161,162,163], he has presented various modelling languages (such as Modelica, Simulink, AADL, MARTE, and SysML) and compared them with different views of the CPS. Furthermore, in several publications, he considered the interactions with Big Data [164,165] and how the different languages can be used together [166,167]. Babris et al. [129] also examined a number of modelling languages for designing CPS and compared them against several criteria. Similar collections of modelling languages and tools can be found in the works of Buffoni et al. [168], Attaerzadeh-Niaki and Sander [169], as well as Staroletov et al. [170].

One of the most frequently considered modelling languages is UML (Unified Modelling Language), or modifications of it. Woo et al. [171] used the UML modification xUML (eXecutable Unified Modelling Language) for modelling CPS as early as 2008. Lichen Zhang used UML for modelling [172] and, in a follow-up work, extended the successor UML2.0 to a Cloud-Based Hybrid UML metamodel (CHUML) [173]. More recent publications increasingly rely on UML2.0 and corresponding modifications. For example, Zhai et al. and Zhou et al. used Hybrid UML, which is a profile of UML 2.0 [174,175]. Sadwovykh et al. and Tannoury et al. both used SysML, which is a graphical standardised modelling language based on UML 2.0 [176,177]. Tannoury et al. further linked SysML with MARTE and OCL (Object Constraint Language), as well as Reo for additional constraints [176]. Wilking et al. [178] utilised SysML to design and operate Digital Twins. Wang et al. [179] described a bidirectional mapping structure between Simulink and UML.

Another modelling language used in the literature is the Architecture Analysis and Design Language (AADL). While Wu et al. [180] used it to model the behaviour from different domains for a CPS, Renya et al. [181] combined AADL with the modelling language Modelica. Sales et al. [135] transferred the results of an ontology analysis into an AADL model. Modelica was also used by Wawrzik et al. [182] in combination with other languages. They presented a simulation framework called SICYPHOS (SImulation of CYber PHysical Systems), which integrates SysML, Modelica, SystemC, and C/C++. Junjie et al. [183], on the other hand, used Modelica alone to model CPS. They contrasted the Modelica features (object-oriented modelling, equation-based modelling, connect-based modelling, and hybrid modelling) with the CPS system modelling approaches (physical system modelling, information system modelling, and interface modelling). Schroeder et al. [138] used the modelling language AutomationML (Automation Modelling Language) for modelling. Centomo et al. [184] linked models of different levels of abstraction of a manufacturing plant. Lehner et al. [185] introduced the AML4DT (Automation Modelling Language for Digital Twin) framework for this purpose. Fitzgerald et al. [186,187] used the Unifying Theories of Programming (UTP) approach and its large-scale application in the form of the definition of the COMPASS Modelling Language (CML) [187].

In addition to the dedicated modelling languages, there are methods in the literature using mathematical modelling approaches. Lee et al. [188] examined various mathematical models for describing discrete event systems (DES) in the context of CPS. One mathematical model considered is Petri Nets, which is used by several other authors. He et al. first used this to model CPS [139], and then extended it to Predicate Transitions Nets (PrTNs) in another publication [189]. Quian and Yu, in particular, used time-constrained aspect-oriented Petri nets (TAOPN) [190].

Several authors used (interconnected) flow and decision diagrams to model the behaviour of the product [191,192]. Christofi and Pucel [193] described the behaviour of a product using fault trees and behaviour trees, while Negri et al. [194] used the identified states (idle, working, error, emergency button, and energy-saving mode) of the product to model it. Doka and Horak [195] created block diagrams of a gearbox to represent and simulate its behaviour. Steinmetz et al. [136] used the application Node-Red to create knowledge graphs of an asset. Meryem Afendi [151] used Event-B to model a CPS. Tou et al. [196] extended the traditional hybrid system description language HYSDEL to E-HYSDEL. This can be used to describe the behaviour of CPS. Janda et al. [197] utilised the methods Virtual Numerical Controller Kernel (VNCK) and Mechatronic Concept Designer (MCD) on a case study and compared the results. Erkoyuncu et al. [198] presented an ontology concept that is used to describe an asset and model the behaviour of the Digital Twin. The focus is on the adaptivity of the Digital Twin. Eyre et al. [116] used CAD data to map the geometric properties of the product in the Digital Twin. Lai et al. [199] also used CAD models to derive a mesh, which was then used to calculate the power flow (Optimal Power Flow—OPF).

There are approaches that make use of special libraries for the model creation. Zhao et al. [200] used a component-based reduced order modelling (ROM) technique to create a Digital Twin of a wind turbine. The components are stored in a library and can be selected and combined from there. Zou et al. [201] presented a process model for a Digital Twin to make statements about the quality of machined parts. They considered key features, which they stored in a library.

Apart from these system-based modelling approaches, there are data-based approaches, which find specific application in the examined literature in the context of machine learning [146]. Lou et al. [148,149], Dashkina et al. [202], as well as Tarkhov and Malykhina [203] used neural networks as behavioural models of a Digital Twin. Liu et al. and Yang et al. further used transfer learning approaches to adapt the models to changing conditions, for example, and thus increase robustness [204,205]. Zheng and Ni [142] also used real data to retrain the parameters of their model, creating a hybrid model.

## 5. Discussion and Need for Research

**Figure 13:**
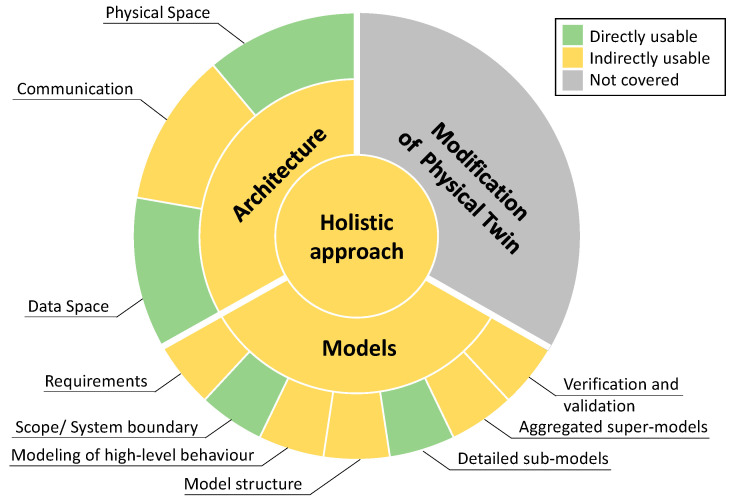
Overview of the usability of the literature on various topics (size of the individual elements has no significance).

Finally, here, the results are compared and discussed with the research questions formulated at the beginning. The first research question (RQ1) deals with the topic of how the creation of Digital Twins, Cyber-Physical Systems, and Product-Service Systems is considered in the literature and what research directions exist. The discussion is visualised in Figure 13. This shows the overarching holistic approaches with the three domains: architecture, models, and modification of physical twins, as well as their respective sub-topics. The individual elements are coloured according to the usability of the literature. Topics that allow direct utilisation of the literature are coloured green, while indirect utilisation is coloured yellow. Topics that are not covered in the literature, or only to a limited extent, are coloured grey. The size of the individual elements has no significance in this illustration.

Several holistic approaches for the creation of Digital Twins and Cyber-Physical Systems can be found in the literature. Existing development approaches were analysed regarding their suitability for the creation of Digital Twins and criteria for this were formulated. Furthermore, approaches were described, which were either a suitable modification of the existing approaches or completely new approaches for the creation of Digital Twins. However, the described procedures were either on a rather superficial and general level or considered only selected sub-steps or even domains in the creation of Digital Twins. The individual steps described in the holistic approaches overlapped or complemented each other to a certain extent. However, it was not possible to adequately cover the domains described in more detail below. One reason for this is that the scope of the holistic approaches greatly differs.

The review of the literature has shown that several domains need to be mastered in order to effectively create Digital Twins. The two most dominant domains in the literature were clearly the models and the architecture. In addition, other domains were also mentioned, although they received less focused attention in the literature. For example, it is necessary to modify the physical product to make it usable with Digital Twins. 

Within these three domains, the focus of the literature is on the models, which are a key aspect of Digital Twins. A large number of publications presented individual steps for creating product-describing models. Since these individual steps have a great degree of overlap, they can be combined into a comprehensive sequence of steps. Two individual steps received special attention in the literature. One step was the model scopes, which help in the identification of the scopes. Here, the authors described which aspects of the physical twin are represented within the context of the Digital Twin. The other step that received special attention in the literature was the specific model types for the creation of detailed sub-models. Various modelling approaches were presented here. Due to this particular focus, these two steps are directly feasible, and the literature is directly usable. The literature on the other steps focused primarily on theory and is only indirectly applicable in a practical sense. Since only some of the steps in context of the models are directly applicable, the literature in this domain can only be classified as indirectly usable, as well.

The second domain, which was also dealt with extensively in the literature, was architecture. This was divided in the literature into physical space, data space, and the communication in between. Most of the literature was limited to the description of this architecture and less to tools, methods, or guidelines to create this architecture. The detailed description of the physical space and the data space nevertheless allow a direct use of the corresponding literature. Users can use the detailed architectures described to help them overcome their own challenges. Nevertheless, specific guidance would be more desirable. The communication between the two spaces mentioned is only treated superficially in the literature and can only be used to a limited extent. Since the subcategories in the field of architecture can only be used partially directly, the literature in this domain can only be classified as indirectly usable.

The last domain mentioned, the modification of the physical twins, was only marginally dealt with in the literature. In particular, there were no systematic procedures for the selection and integration of sensors. This is marked by the grey colour in Figure 13. For this reason, a systematic literature search for sensors in the context of Digital Twins and CPS was carried out in parallel to this contribution; however, the results were not discussed here, and instead reference is made to the corresponding contribution [206]. There is a need for further research on actuators in the physical twin.

For a holistic approach, all three domains must be interconnected and the interactions between them must be considered. This was only partially covered in the literature on holistic approaches. The indirect usability of the literature in all three domains does not allow a direct link to a holistic approach.

The second research question (RQ2) addresses the question of in which domains and to what extent further research is necessary. An answer to this question can also be derived from the previous discussion of the research results.

There is a need for further research in the domain of architecture. In particular, the description of communication cannot be directly applied in practice. Further research and documentation of the results is necessary here. Furthermore, there are no recommendations for action or lists of necessary procedure steps for the creation of a suitable architecture for a Digital Twin in the literature. This is a key need for future research.

The domain of modelling is described in the literature as specific action steps. As described in Section 4.3.1, these can be grouped into a unified procedure. However, there is still a need for further research. The literature that describes the individual steps can often only be used indirectly. More research is needed, especially in the areas of requirements, modelling of high-level behaviour, model structure, aggregated super-models, and verification and validation.

The modification of the physical twin was hardly covered in the literature. This includes, for example, the selection and integration of sensors and actuators in the product, and it represents a significant need for research. For this reason, as already mentioned above, a systematic literature search on the topic of the selection and integration of sensors for Digital Twins was carried out parallel to this contribution [206]. The results are rooted in the work of Hausmann et al. [207,208] on sensor integration. Comparable research in the field of actuators is still needed.

Finally, further research is also needed in the area of holistic approaches. In the literature, only a few aspects of a few domains are currently considered. Based on the results of the discussed domains, the consideration of all domains and, in particular, the linkage and interactions between them, is a subject for future research. Furthermore, the presented procedures are all at a moderate level of abstraction. This degree of abstraction can be varied during further research. An increase in the degree of abstraction would lead to a consideration of the partial model level or the RFLP model, which would enable a holistic view and allow the identification of systematic errors or blind spots. Reducing the level of abstraction would transform the results into specific guidelines.

## 6. Conclusions and Outlook

In this contribution, a systematic literature review on the development and creation of Digital Twins, Cyber-Physical Systems, and Product-Service Systems was conducted. A total of 185 articles were identified and examined. With this, the first research question (RQ1), formulated in the Introduction Section, of how the creation of Digital Twins, Cyber-Physical Systems, and Product-Service Systems is considered in the literature, was answered. Furthermore, the contents of the articles were clustered and divided into three categories: “holistic approaches”, “architecture”, and “models”, and their corresponding subcategories. From this, two domains were identified that are necessary for the Digital Twin: models and architecture. A more detailed analysis showed that sensors and actuators are also necessary, which is a third necessary domain. However, this was not sufficiently covered in the literature, as found during the research in this contribution. In the domain of models, there is a large number of literature articles describing procedures at different levels of granularity. There are specific procedure steps described for modelling. Furthermore, abstract model scopes or models are described, but also specific model types for specific use cases. In the domain of architecture, the literature is limited to the description of the physical space, the data space, and the network. Specific components or necessary development steps are not mentioned. The dependencies and interactions between the domains are also not considered in the literature. These aspects represent clear research gaps and require further research (RQ2).

## Figures and Tables

**Table 2:** Definitions of the terms: architecture, framework, and IT infrastructure.

Term	Definition	Source
Architecture	“Architecture is a unified structure for the purpose of implementing a technology. It can be used to decompose technology into key elements and help to integrate them into existing or new ecosystems with minimal efforts”.	[82]
Framework	“A framework is a semi-complete application. A framework provides a reusable, common structure to share among applications. Developers incorporate the framework into their own application and extend it to meet their specific needs. Frameworks differ from toolkits by providing a coherent structure, rather than a simple set of utility classes”.	[83]
IT Infrastructure	“IT infrastructure is the system of hardware, software, facilities and service components that support the delivery of business systems and IT-enabled processes”.	[84]

## Data Availability

Data are contained within the article.
